# Encountering Suffering at Work in Health Religious Organizations: A Partial Least Squares Path Modeling Case-Study

**DOI:** 10.3389/fpsyg.2020.01424

**Published:** 2020-06-23

**Authors:** Maria Isabel Sánchez-Hernández, Eduardo Gismera-Tierno, Jesus Labrador-Fernández, José Luis Fernández-Fernández

**Affiliations:** ^1^Business Management and Sociology Department, School of Economics and Business Administration, University of Extremadura, Badajoz, Spain; ^2^Ethics and Sustainability Department, School of Economics, Comillas Pontifical University, Madrid, Spain

**Keywords:** health, hospitals, quality, nurse-patient relationship, religious organizations, suffering

## Abstract

Health religious organizations tend to offer individual attention to patients in line with their spiritual character and, at the same time, the highest service quality. This study puts the attention on the nurse-patient relationship and empirically explores a theoretical model that links nurses’ suffering at work with personal’s willingness to engage in a therapeutic and spiritual relationship with patients and the consequent effect on quality. Data has been collected in the city of Madrid (Spain) in the month of June 2019 in Santa Elena Clinic. An analytical case-study based on Partial Least Squares (PLS) path modeling is the chosen method to verify the cause-effect hypothesized relationships. This study contributes to the current academic literature by providing new knowledge and empirical evidence on the topic of the future of work in health religious organizations. The main conclusion is the necessary inclusion of suffering, even in good places to work, as a key indicator for a better management. Results should be a useful source of information for practitioners that seek to implement better human management systems in these organizations.

## Introduction

Poor knowledge exists concerning the effects of suffering at work on health and occupational safety (Tetrick and Quick, 2003). Although researchers are becoming increasingly interested in understanding suffering at work such as Gismera et al. (2019), and companies start to be conscious about the organizational problems associated is an unexplored topic. The current human management systems do not directly address this problem, and there is a lack of evidence regarding how to manage suffering in organizations.

Religious health care institutions, most of them Catholic in Spain, are communities of healing, but also communities of compassion. That means that the service offered is not limited to the clinical treatment of the disease (physical dimension). In addition, religious health care institutions embrace the psychological, social and spiritual dimensions of the patients and the medical expertise is combined with spiritual forms of care to minimize human suffering. The encounter of Christians with human suffering is taken on a positive meaning through the redemptive power of Jesus’ suffering and death. At this respect, the United States Conference of Catholic Bishops (2001) highlighted “healing and compassion as a continuation of Christ’s mission and suffering as a participation in the redemptive power of Christ’s passion, death, and resurrection.” Going beyond the achievement of service quality, that is a general business goal (Lee et al., 2000; Glickman et al., 2007) to develop a close relationship with patients is considered the central aspect of health religious organizations. Consequently, measuring and managing the suffering in the workplace seems to be more important in this type of organizations, devoted to mitigate the suffering of others from the physical and the spiritual dimensions, than in other non-religious.

This study explores a structural model that links suffering at work with personal’s willingness to engage in a therapeutic and spiritual relationship. Data was collected in Madrid in the month of June 2019 in Santa Elena Clinic. Partial Least Squares (PLS) path modeling is the chosen analytical method to test the model and identify cause-effect relationships. This study will contribute to the academic literature by providing new knowledge and empirical evidence on the topic and a very useful source of information for practitioners that seek to implement better human management systems in health religious organizations.

## Theoretical Framework

Suffering at work has been recently defined as a destabilizing and unpleasant psychological experience arising when the employee runs into insuperable and tenacious barriers, after having used up all his or her available resources trying to improve the organization of work with regards to quality and safety (Bontemps et al., 2018). According to the Psychodynamics Theory (PT) of work and organizations, a person’s working experience undergoes the same intrapsychic conflicts as other aspects of life. Czander (1993) pointed out that the traditional view of system man/woman is that he/she is motivated by the need for material goods (economic matters) but the psychoanalytic perspective defends the neurotic condition of all human beings conditioned by shadowy wishes and fears. Under this theory, Diamond and Allcorn (2003) defined organizations as processes of human behavior that are considered as experiential and perceptual systems dominated by unconscious schemes (Prins, 2006). The workplace has been conceptualized as psychological space and a meaningful workplace implies a good relationship employee-organization in terms of dedication, loyalty and commitment (Chalofsky, 2003; Glomb et al., 2011) where spirituality has a relevant role (Steenkamp and Basson, 2013). Some recent works exist devoted to the study of these topics within the context of religious organizations (Ariza-Montes et al., 2017, 2019; Ortiz-Gómez et al., 2020).

Most human beings are inclined to avoid suffering (Connelly, 2009). The recommendation of Diamond and Allcorn (2003) for human resources managers is to bring into the workplace those things that contribute to making work a meaningful experience that will contribute to the employees’ mental health. In the same vain, and following some authors from the field of coaching such as Briner (1999), de Vries (2004), Peltier (2011) in this research the argument is made that unconscious dynamics related to job satisfaction, but also suffering at work, causes a significant impact on organizations and, in addition, on service quality (Ellinger et al., 2011).

Job satisfaction is a complex and multifaceted construct (Judge and Kinger, 2007). Locke (1976) defined job satisfaction as a “pleasurable or positive emotional state resulting from the appraisal of one’s job or job experiences.” In general terms, job satisfaction is reflected on the positive perception of professional achievement (Gazioglu and Tansel, 2006) related to motivation (Tzeng, 2002; Egan et al., 2004), spiritual well-being (Duggleby et al., 2009) psychological well-being (Munir et al., 2012; Burton et al., 2017), recognition (Ernst et al., 2004; Clarke and Mahadi, 2017), and pride for a given work activity (Utriainen and Kyngäs, 2009). It has been also recognized that satisfaction at work is reflected in the opportunity to express feelings and opinions to colleagues and supervisors and different experiences at work such as solidarity, cooperation or the possibility of using creativity at work (Noon et al., 2013; Guillén et al., 2015). In this context, although job satisfaction in the health care field is considered a significant element of health service quality (Snipes et al., 2005; Mosadeghrad, 2014; Choi et al., 2016), it is still largely understudied.

In general terms, traditionally medical literature has contained few studies that specifically address suffering (Cassell, 1982) and most authors who have tried to define suffering argued for its complementarity with the term pain (Egnew, 2005). However, the study of human suffering has advanced considerably in the last years (Coulehan, 2018). Some authors such as Montoya-Juarez et al. (2013) defined suffering as a state of pain more or less permanent, experienced by the subject within a specific society and culture, when facing a perceived threat as capable of destroying their own physical or psychosocial integrity, and before which they feel vulnerable and helpless.

Suffering in the workplace is probably a more common occurrence than expected in everyday life. As individuals of the first quarter of the 21st century, we spend much of our time around the professional side, in the bosom of business organizations of one kind or another and of the most diverse sectors. The suffering of human beings can not be subtracted from us. In fact, suffering at work represents a condition that drives worker mobilization against inconsistencies experienced at work and may even trigger mental disease.

It coul be said that the workplace itself is a source of suffering. Probably, this is more accurate in specific professions in front-office, assuming risks and/or dealing with high responsibilities. Suffering at the workplace can be evaluated through the experience of exhaustion that is caused by the perception of stress (Quick and Henderson, 2016) dissatisfaction (Häggström et al., 2005), insecurity or fear (Probst and Lawler, 2006; Cheng and Chan, 2008).

However, that is a fact in all sectors and job positions when the increase of competition and the derived new forms of work organization are even causing harm employees’ mental health (Hirigoyen, 2008). At this respect, Quick and Henderson (2016) reviewed the evidence concerning the health risks associated with occupational stress. Their arguments and final conclusions, that were developed from roots in preventive medicine and public health, highlighted the application of preventive management. In the interests of both, employee well-being and company performance, suffering at work must be taken seriously by Human Resources Managers, especially in the service sector, because it is expected that it will impact the final quality of the service provided.

Moving to the specific reality of suffering at work in staff nurses, the work of Elpern et al. (2005) about moral distress in a medical intensive care unit is a good example. The author identifies frequent and intense contexts where nurses suffer at work dealing with specific treatments for terminally ill patients and related technical and moral/ethical decisions. In academic literature, other authors have analyzed nursing and suffering at work focusing the attention on work stress (Taylor et al., 1999; Adeb-Saeedi, 2002; Dominguez-Gomez and Rutledge, 2009) burnout (Malach-Pines, 2000; Alimoglu and Donmez, 2005; Pompili et al., 2006; Khamisa et al., 2013) or both, in relation to the environment and the working conditions (Jennings, 2008).

Service quality has been deeply studied in academic literature originally from the Nordic School (Chowdhary and Prakash, 2007; Gummesson et al., 2012) and especially in health services (Isaac et al., 2010; Kitapci et al., 2014; Meesala and Paul, 2018). Once the existence of suffering at work in organizations has been recognized, it must be managed in health religious clinics and the quality of the healthcare service must be guaranteed.

To sum up, satisfied personnel in religious health-care organizations would develop a trusting and connected relationship with patients. Thus, we can expect high levels of nurse-patient relationship (Moyle, 2003; Mok and Chiu, 2004; Ward et al., 2009) and high levels of service quality (Landrum et al., 2009). However, poor knowledge exists concerning the effects of nurses’ suffering at work on service quality, basically because the current human management systems do not directly address this problem. Trying to contribute to fill the gap, this study contributes to the topic with new insights.

## Materials and Methods

### Design

An analytical case-study based on primary data was carried on. The case-study had three differentiated steps. Firstly, the Santa Elena Clinic was selected for being considered a best place to work. To verify this assumption nurses’ satisfaction at work was measured throughout an *ad hoc* anonymous self-administered questionnaire.

Secondly, the study analyzed the causal effect between nurse-patient relationship and the quality of the service provided (Model A). Finally, suffering at work was included to test the effect on the process of attending the patients and attaining the goal of high service quality (Model B).

**FIGURE 1:**
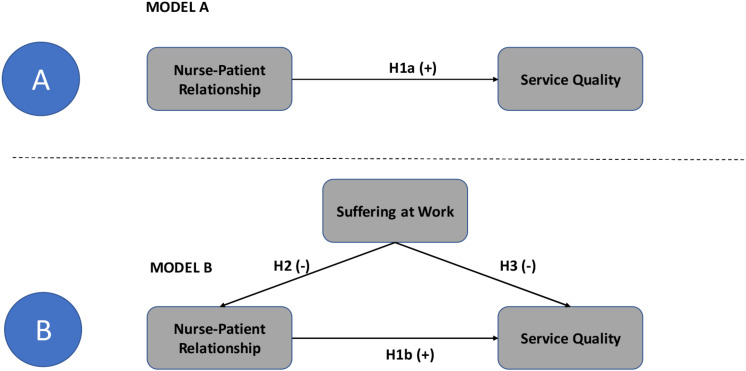
Theoretical Models **(A,B)**.

Figure 1 shows the theoretical model A and B on the basis of this study representing the following hypotheses:

H1: Nurse-Patient Relationship has a direct and positive impact on service quality.

H2: Suffering at work has a direct and negative impact on nurse-patient relationship.

H3: Suffering at work has a direct and negative impact on service quality.

### Participants and Procedures

Catholic Hospitals of Madrid is the first non-lucrative health group in the city of Madrid in Spain. One of its members is Santa Elena Clinic, a Catholic health organization that has been run for more than half a century by the Religious Society of Saint José from Gerona. In all the centers the patient receives an individual attention in line with the human and spiritual character of the religious organization based on the maximum respect for the person and for life. This work is carried out with high level of dedication and love by religious, volunteers and professionals.

A total of 140 nurses were contacted in Santa Elena Clinic in Madrid, in the month of June 2019. A pilot test was carried out in order to check that the survey would be appropriately interpreted by the respondent. A final sample of 102 valid questionnaires were collected. Table 1 presents the study’s technical data sheet.

**TABLE 1:** Technical data sheet.

Geographical Scope	Madrid (Spain)
Method of information collection	Personal contact
Population	140 nurses
Final sample	102 nurses
Index of participation	73%
Measurement error	5%
Trust level	95% *z* = 1.96 *p* = *q* = 0.5
Sampling method	Simple random
Average duration	3:40 (minutes: seconds)

### Data Analysis Methods

Descriptive statistics first, and structural equations modeling (SEM) later, have been used considering it very suitable for our research interests following Czander (1993). Concretely we want to highlight two reasons: because the constructs under study and the relationships between them, are really new; and both, the theoretical model and the relative measures, are not very well formed and generally accepted in the academic literature.

Structural equations modeling has been selected as the appropriate methodological tool considering that it offers the possibility to combine and compare theory with empirical data, performing multiple regressions between several latent variables to provide causal scientific explanations that go beyond the description and the association. In the present study, it has been applied the software developed by Ringle et al. (2005) denominated Smart-PLS (Partial Least Squares). PLS focuses on the explanation of variance using ordinary least squares, this technique is suited for the investigation of relationships in a predictive rather than a confirmatory fashion (Fornell and Bookstein, 1982). As a multivariate variance-based technique, PLS estimates path models involving latent constructs indirectly observed by multiple indicators. Furthermore, PLS is not sensitive to the assumptions of normality.

After verifying high levels of job satisfaction in the nurse-staff, a step-by-step analysis was conducted to offer a thorough analysis. In the first step, the focus was on the relationship between nurse-patient relationship and service quality. Subsequently, suffering at work was introduced, and the full structural model was assessed.

### Measures and Instrument

An *ad hoc* questionnaire was elaborated to inquire into the nurse’s perceptions concerning different constructs, with responses on a 5-point Likert scale. According to previous literature review, satisfaction at work was measured with the following six items: Satisfaction with professional achievements (S1); Proud to work here (S2); Freedom of expression to both, colleagues and bosses (S3); Trust in co-workers (S4); Feelings of solidarity and cooperation at work (S5); Possibility to be creative at work (S6). Nurse-patient relationship was measured throughout four items: Understanding the patients’ needs (NPR1); Displaying caring actions and caring attitudes (NPR2); Providing holistic care (NPRN3); Acting as the patient’s advocate (NPR4). Service Quality was measured throughout two items, Affective and therapeutic dimensions: Willingness to listen patients and to assist them to talk about their fears, anxieties and problems (SQ1); Willingness to help patients and provide prompt service (SQ2). Finally, suffering at work was measured with a direct and single item; Fear and insecurity at work (SFF).

### Ethical Considerations

Health is a matter of public interest. Putting the focus on human resources management in health institutions, the study has been guided by considerations of social justice and decent work, seeking for sustainable job positions and guaranteeing the agreement of managers to perform the analysis and, at the same time, the anonymity for participants.

## Results

After verifying 3.9 average on job satisfaction on the sample, we considered the validity of the scales and the reliability of the measurement Model A and B (the *inner models*). The purpose was to analyze whether the theoretical concepts were properly measured by the observed items. This analysis was carried out for the two attributes validity (measuring what one really wanted to measure) and reliability (whether the process is stable and consistent). To this end we proceeded to calculate the reliability of individual items, the internal consistency or reliability of the scales and the analysis of the average variance extracted obtaining satisfactory results (Table 2).

**TABLE 2:** Main results.

AVE		Composite reliability	*R*^2^	Fornell-Larcker			Heterotrait-		
				
				criteria			Monotrait		
				NPR	SQ	SUFF	NPR	SQ	SUFF
NPR	0.53	0.81	0.08	0.73					
SQ	0.57	0.72	0.17	0.41	0.75		0.83		
SUFF	1.00	1.00		–0.28	–0.12	1.00	0.33	0.20	

**FIGURE 2:**
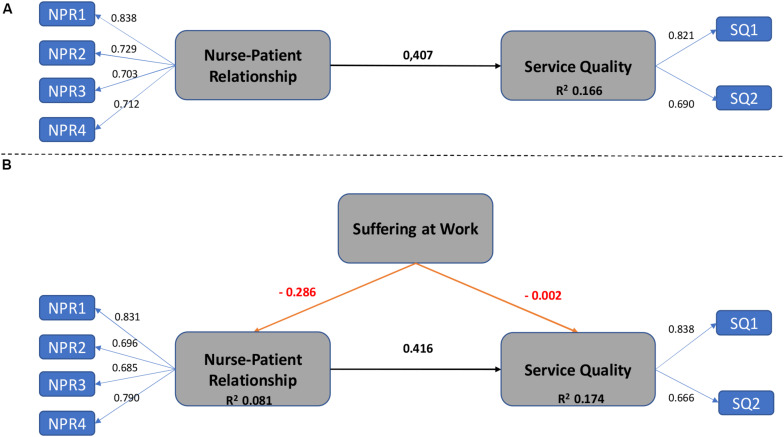
Main results: Models **(A,B)**.

After the satisfactory evaluation of the measurement models it was necessary to carry out a correct interpretation of the structural models in order to verify whether these models considered the proposed relationships between the latent variables. A structural model estimates the assumed causal and linear covariance relationships among exogenous and endogenous latent constructs. Figure 2 shows graphically the main results from Models A and B.

To conclude the analysis, Table 3 shows the hypotheses testing after bootstrap procedure. It was confirmed that the nurse-patient relationship is positively related to service quality but the consideration of suffering at work reduce the positive impact. At the same time, it was verified the significant but negative relation between suffering and both, the nurse-patient relationship and the service quality as was previously hypothesized.

**TABLE 3:** Hypotheses testing.

**Hypothesis: A → B**	**Original path coefficient (β)**	**Mean of sub- sample path coefficient**	**Error**	***t*-value**
***H*_1:_** NPR → SQ	0.41594	0.41520	0.01386	30.011***
***H*_2:_** SUFFERING → NPR	–0.28523	–0.28527	0.01548	18.420***
***H*_3:_** SUFFERING → SQ	–0.12057	–0.11934	0.01463	8.2372***

***p < 0.001 (based on a Student’s two-tailed test, *t*_(499)_) *t*_(0.05;499)_ = 1.96; *t*_(0.01;499)_ = 2.59; *t*_(0.001;499)_ = 3.31.

## Discussion and Conclusion

Health religious organizations offer professional and spiritual attention to their patients but suffering at the workplace exists, and often at the cost of service quality. In this work the nurse-patient relationship has been empirically explored through two complementary models. The first hypothesis of the study was verified in Model A when testing the direct and positive link between nurse-patient relationship and service quality. The results obtained from the second model, Model B, served for a twofold purpose.

On the first hand, it confirms that suffering at work exists even in good places to work for, and even in religious organizations such as Santa Elena Clinic, with high levels of job satisfaction in their staff. That is in line to the consideration of suffering in the workplace as a more common occurrence than expected. On the second hand, results from Model B confirm previous works defending that suffering causes a significant impact on organizations (Briner, 1999; de Vries, 2004; Peltier, 2011). In our case, and confirming our hypotheses, there is a negative impact of suffering on nurse-patient relationship and also on service quality.

We can conclude that health-care personal in religious clinics, will have difficulties in meeting the needs of their patients and providing the best service to them if their own needs are not previously met as well. The novelty of this work is the study of suffering at work in a specific institution where, *a priori*, nurse-patient interaction is spiritual and therapeutically good, job satisfaction of nurses is high, and the organization presents high service quality standards. Even in this situation, suffering at work appears and negatively impacts both constructs, nurse-patient relationship and service quality. Thus, human resources responsible in healthcare institutions have to start considering measuring and managing suffering at work in order to improve the places to work for and the quality of their care systems.

The results of this research contribute to the literature on suffering at work among nursing staff in healthcare religious organizations by enhancing the understanding of its influence on the nurse-patient relationship and the final health service quality. This study offers new policy insight for healthcare human resource managers who seek to increase service quality among their nursing staff. Managers grasping the importance of suffering at work affecting the well-being of nurses will be more likely to gain improved service quality.

Some recommendations in terms of the Human Resources Management policies in health religious organizations emerge. Assuming that behavior is often the result of conscious and unconscious mental processes, suffering at work must be taken into account when managing personnel to guarantee the quality of the final service. In line with, it should be highly innovative the implementation of questionnaires. The first reason should be accepting a visibility tool for managing suffering. The tool will measure the potential imbalance between what the nurse has to do and what is receiving from its immediate environment causing suffering.

Secondly, the implementation of an appropriate system to measure suffering at work will serve for understanding the phenomena, and for helping nurses to reflect on their emotions when attending patients in order to improve the final healthcare quality system. In the same line, other highly recommended tool according to literature review should be the implementation of mindfulness programs at work (Glomb et al., 2011) because mindfulness training has been shown to be effective in reducing suffering and enhancing psychological wellbeing (Burton et al., 2017) because health religious organizations must be “healthy organizations” as well.

## Data Availability Statement

The raw data supporting the conclusions of this article will be made available by the authors, without undue reservation.

## Ethics Statement

Ethical review and approval was not required for the study on human participants in accordance with the local legislation and institutional requirements. Written informed consent from the participants was not required to participate in this study in accordance with the national legislation and the institutional requirements.

## Author Contributions

MS-H searched and reviewed the study, analyzed the data, wrote the manuscript, and supervised research process. EG-T performed the data collection. EG-T, JL-F, and JF-F critically reviewed the manuscript. All authors contributed to data interpretation and approved the final version of the manuscript for submission.

## Conflict of Interest

The authors declare that the research was conducted in the absence of any commercial or financial relationships that could be construed as a potential conflict of interest.
